# Supplementary material for: Meta-Analysis and Gene Set Enrichment Relative to ER Status Reveal Elevated Activity of MYC and E2F in the “Basal” Breast Cancer Subgroup
Source: PLoS One. 2009 Mar 9;4(3):e4710. doi: 10.1371/journal.pone.0004710 (PMC2650420; doi:10.1371/journal.pone.0004710)
Supplement: Dataset S1 — GSEA results from first screen. The file contains a zipped archive of the HTML results of the second GSEA screen. Reports for enriched or depleted datasets may be accessed from the “index.html” link within the my_analysis.GseaPreranked.1219975950625.rpt.zip ZIP archive. Enrichment plots are available for each of the 50 most enriched and depleted gene sets. (13.32 MB ZIP) [file pone.0004710.s001.zip › How to access the GSEA results.doc]

### How to access the GSEA results:

The results of the analysis of gene sets from the MolSigDB enriched in ER negative (ER-) tumors or in ER positive tumors (ER+) may be found in the following zip archive:

my_analysis.GseaPreranked.1219975950625.rpt.zip

No outside gene sets have been added to the analysis.

The results of the analysis can be accessed by clicking on the **index.html** link, which will take you to spreadsheets of the results.

Gene sets enriched in ER- tumors are indicated by “gene sets are upregulated in phenotype **na_pos”**.

Gene sets enriched in ER+ tumors are indicated by “gene sets are upregulated in phenotype **na_neg”**.

**Finding out about a gene set of interest**

For example, we may interested in finding out about gene sets that are positively correlated with ELF5 expression.

Click on the following link in the na_pos section of the screen.

- Detailed [enrichment results in html](../../../../C:%5CDocuments%20and%20Settings%5Cmarall%5CDesktop%5CElf%5CMar26%20GSEA%20Elf5%20Basal%20Wang%20220625_s_at%5Cmy_analysis.GseaPreranked.1206501611265%5Cgsea_report_for_na_pos_1206501611265.html) format

You will be taken to the following screen:

It appears that a gene set called “BRCA_ER_NEG” is most highly enriched in genes that are enriched in ER- tumors (Normalised Enrichment Score, NES = 3.66, FDR=0.000).

You can learn more about the “BRCA_ER_NEG” gene set by clicking on its hyperlink in the “GS Follow link to MSigDB” column which will take you to the information stored in the “Molecular Signature Database”.

So, the “BRCA_ER_NEG” gene set contains “Genes whose expression is consistently negatively correlated with estrogen receptor status in breast cancer - higher expression is associated with ER negative tumors” in the study of V'ant Veer et al.

The next step would be to find out the genes within the “BRCA_ER_NEG” gene set that are contributing to the high enrichment score. Go back to the original GSEA results page, and click on the “Details” link for this gene set in the GS Details column.

This gives the enrichment plot – Profile of the Running ES Score & Positions of Gene Set Members on the Rank Ordered List. Gene set members are indicated by black bars. We can see from this plot that the majority of members of this gene set are towards the top of the list of genes ranked by their correlation with ER- status, and there are few members with negative correlation.

Scroll further down this page to see the genes within this gene set contributing most to the high enrichment score (indicated by a “Yes” in the “Core enrichment” column).
